# The Potential Future Role of Genetic Markers, Neurophysiological Insights, and AI Innovations in Personalized Attention-Deficit/Hyperactivity Disorder (ADHD) Management

**DOI:** 10.7759/cureus.93949

**Published:** 2025-10-06

**Authors:** Vimal Satodiya, Surendra Gupta

**Affiliations:** 1 Psychiatry, Inland Psychiatric Medical Group, Lancaster, USA; 2 Pediatrics, Clinica Sierra Vista, Fresno, USA

**Keywords:** adhd, biomarkers, eeg-based motor-pattern analysis, sl6a3, tbr

## Abstract

Attention-deficit/hyperactivity disorder (ADHD) is a complex neurodevelopmental disorder marked by inattention, hyperactivity, and impulsivity, with significant genetic and environmental influences. Its heritability rate is estimated with genetic markers such as *SLC6A3*, *DRD4*, *ADRA2A*, *COMT*, *DRD5*, and *SLC6A2* influencing dopamine and norepinephrine regulation. These markers may impact ADHD risk, subtypes, and treatment responses, potentially enabling pharmacogenetic insights into stimulant efficacy and adverse effects. Neurophysiological biomarkers, particularly EEG-derived theta/beta ratio, may enhance diagnostic precision by identifying ADHD-specific cortical activity. Quantitative EEG (qEEG) and biofeedback further aid in treatment monitoring. Non-invasive biomarkers, including magnesium and zinc deficiencies, inflammatory cytokines like interleukin-6 (IL-6), and salivary markers, such as cortisol, alpha-amylase, and secretory immunoglobulins, expand ADHD diagnostic tools. Artificial intelligence (AI) may revolutionize ADHD management in the future with scalable tools like EEG-based motor analysis, eye-tracking systems, and wearable devices. AI-powered models, including socially assistive robots and hybrid algorithms, may enhance engagement and personalize therapy, particularly in underserved regions. Despite advancements, challenges remain in standardizing biomarkers, validating AI tools, and addressing ADHD heterogeneity. Future research should integrate genetic, neuroimaging, and AI-driven multi-modal approaches to improve diagnostics and optimize therapies. Holistic interventions, such as pharmacogenetics and magnesium supplementation, show promise for enhancing ADHD care. By uniting genetic, neurophysiological, and technological insights, these innovations pave the way for precise, accessible, and personalized ADHD management strategies.

## Introduction and background

Attention-deficit/hyperactivity disorder (ADHD) is a neurodevelopmental disorder that has drawn significant attention since its early recognition in the 20th century. First described by Sir George Frederick Still in 1902 [1], ADHD has evolved from being identified as "hyperkinetic impulse disorder" to its current classification in the Diagnostic and Statistical Manual of Mental Disorders (DSM-5) [2], which recognizes three presentations: predominantly inattentive, predominantly hyperactive-impulsive, and combined [3]. This evolution reflects advances in understanding ADHD as a complex condition influenced by genetic, neurobiological, and environmental factors [4].

Globally, ADHD affects approximately 5% to 7% of children, though regional variations occur, with prevalence rates as high as 15% in certain populations [5]. Globally, prevalence varies widely, with rates as high as 15% in regions like the Middle East and as low as 2% in countries like Finland, reflecting differences in diagnostic practices and cultural perceptions [6]. Variations in prevalence highlight the role of cultural perceptions, diagnostic criteria, and healthcare access, underscoring the importance of tailored intervention strategies [7].

Recent research into ADHD etiology has highlighted genetics and pharmacogenetics, biomarkers, and artificial intelligence (AI) as novel areas that hold promise for advancing diagnosis and treatment. Genetic studies reveal a heritability rate of 70% to 80%, with specific genes involved in dopamine regulation contributing to ADHD risk [8]. Neurotransmitter imbalances, particularly in dopamine and norepinephrine pathways, are central to ADHD's pathophysiology, affecting attention regulation and impulse control [9]. Genetic predispositions can also interact with environmental influences such as prenatal toxin exposure and perinatal complications, further exacerbating the disorder [10]. Advances in pharmacogenetics have begun to elucidate how genetic variability influences individual responses to ADHD medications, paving the way for personalized treatment approaches [11].

Biomarker research represents another burgeoning field, offering objective diagnostic tools that transcend subjective assessments. Neuroimaging studies have revealed structural and functional abnormalities in the prefrontal cortex and other brain regions linked to ADHD symptoms [12]. Emerging biomarkers, including pupil dynamics, EEG patterns, and eye-tracking metrics, are providing quantifiable insights into ADHD's neurobiological underpinnings, potentially enhancing diagnostic accuracy and treatment monitoring [13].

Artificial intelligence (AI) is also transforming ADHD research, leveraging machine learning algorithms to analyze complex datasets and improve diagnostic precision. AI-powered tools, such as wearable devices and eye-tracking technologies, offer scalable and accessible solutions for identifying ADHD-specific patterns [14]. Additionally, AI has been applied in therapeutic contexts, using digital platforms and robotics to enhance patient engagement and tailor interventions. These advancements may address barriers associated with diagnostic delays, clinician shortages, and subjective assessments, particularly in resource-limited settings, though further validation is needed [15].

This review aims to explore the interplay of genetics and pharmacogenetics, biomarkers, and AI in ADHD, examining their potential to refine diagnostic and therapeutic strategies. By integrating insights from these three novel areas, it seeks to provide a comprehensive perspective on how emerging technologies and scientific advancements can optimize ADHD care, enabling more precise, personalized, and accessible interventions.

ADHD is a complex, multifactorial neurodevelopmental disorder characterized by significant clinical and genetic heterogeneity. Genetic factors are pivotal in understanding ADHD’s pathophysiology, and research has consistently pointed to the heritable nature of the disorder. Studies exploring ADHD genetics, including family studies, twin studies, molecular genetic research, and large-scale genome-wide association studies (GWAS), have provided substantial evidence for the role of genetics in ADHD [16,17]. Additionally, advances in linkage disequilibrium score regression and structural equation modeling have allowed for a more precise understanding of ADHD’s genetic correlations with other psychiatric disorders, advancing novel approaches to its treatment and diagnosis [18].

A detailed search was conducted in PubMed, EMBASE, and PsycINFO (2010-2023) using keywords: ADHD, genetics, pharmacogenetics, biomarkers, and AI. Inclusion criteria were peer-reviewed studies in English; case reports and non-human studies were excluded. Meta-analyses and randomized controlled trials were prioritized.

## Review

Heritability and genetic research in ADHD

Family-based and twin studies have been fundamental in identifying the hereditary basis of ADHD. GWAS studies estimate that common genetic variants account for about 22% of ADHD heritability, with individual markers (e.g., DRD4, SLC6A3) contributing modestly (1-3% per allele). Studies have demonstrated that first-degree relatives of individuals with ADHD have a 4- to 5-fold increased risk of developing the disorder compared to the general population [19,20]. This finding was supported by further research that highlighted familial clustering of ADHD, emphasizing its genetic component [21]. These studies laid the groundwork for the genetic exploration of ADHD, confirming its substantial heritability.

Building upon these insights, twin studies have provided even stronger evidence for ADHD’s genetic basis. A population-based registry study, which included over 1.6 million individuals, revealed that monozygotic (MZ) twins had a 70-fold higher risk of ADHD compared to the general population, while dizygotic (DZ) twins exhibited a risk similar to full siblings (hazard ratio: 8.27) [22]. These findings underscore the importance of genetic relatedness in ADHD familial aggregation, with full siblings and MZ twins demonstrating the highest risks. Furthermore, the study noted the influence of shared environmental factors. Maternal half-siblings exhibited higher risks (HR: 8.27 vs 2.34), which suggests prenatal environmental factors, including maternal stress, that may interact with genetic risk [22].

Genetic correlations with comorbidities

Recent research has also explored genetic correlations between ADHD and other disorders. One large cohort study based on Swedish national registries revealed that ADHD is genetically associated with autoimmune diseases (ADs), with an adjusted odds ratio (OR) of 1.34 for affected individuals [23]. This study highlighted the shared genetic risk factors between ADHD and ADs, suggesting that these disorders may not only co-occur in families but also share common underlying genetic mechanisms. It also questioned prior hypotheses regarding maternal immune activation, proposing that genetic risk factors, rather than environmental influences, contribute to this association.

Additionally, studies investigating ADHD's comorbidities with conduct disorder (CD) have revealed the interplay between genetic and environmental factors. Research involving ADHD probands and their relatives demonstrated a significantly higher risk for both ADHD and antisocial personality disorders among relatives of probands with ADHD and CD [24]. This finding suggests that ADHD with comorbid CD may represent a distinct familial subtype, further supporting the genetic basis of ADHD in the context of comorbid disorders.

Novel approaches and ADHD genetics

Emerging research is beginning to link ADHD genetics with specific behaviors and other comorbid conditions. One study identified a significant association between the risk allele C* of the non-coding RNA marker rs1329650 (LOC100188947) and ADHD diagnosis, particularly in individuals with heightened behavioral issues such as aggression and poor executive function [25]. This allele may contribute to ADHD risk and comorbid behaviors through shared genetic mechanisms, offering a novel approach to understanding how genetic variations may drive ADHD and its associated traits.

As shown in Table 1, by identifying specific genetic markers, this research opens avenues for personalized treatments and interventions targeting genetic risk factors for ADHD and its comorbidities. ADHD’s complex genetic architecture, evidenced by family studies, twin research, and molecular genetics, underscores its multifactorial nature. The interconnectivity between ADHD and other psychiatric and medical disorders highlights the genetic risk factors shared across conditions. Advances in genetic research, including the identification of specific markers and the exploration of genetic correlations with comorbidities, offer novel approaches to understanding ADHD and its treatment. These developments may eventually lead to more precise diagnostic and therapeutic strategies based on individual genetic profiles.

**Table 1:** Genetic key observations and findings in ADHD ADHD: Attention-Deficit/Hyperactivity Disorder

Aspect	Key Findings/Results	References	Ref no
Heritability of ADHD	First-degree relatives of individuals with ADHD exhibit a 4- to 5-fold increased risk compared to the general population.	Biederman et al., 1992	[20]
	Familial clustering confirms a strong genetic component (heritability: 70%–80%), with monozygotic twins showing a 70-fold higher risk.	Faraone et al., 2000; Chen et al., 2017	[21,22]
	Maternal half-siblings have higher ADHD risk (HR: 8.27) than paternal half-siblings, suggesting shared prenatal environmental factors.	Chen et al., 2017	[22]
Genetic Comorbidities	ADHD genetically correlates with autoimmune diseases (adjusted OR: 1.34), indicating shared genetic risk factors.	Hegvik et al., 2022	[23]
	ADHD with comorbid conduct disorder (CD) represents a distinct familial subtype, with higher risks of antisocial personality disorders in relatives.	Biederman et al., 1992; Faraone et al., 2000	[20,21]
Genetic Markers	The risk allele C* of rs1329650 (LOC100188947) is associated with ADHD diagnosis, particularly in individuals with aggression and executive dysfunction.	Thakur et al., 2012	[25]
Implications for Therapy	Common genetic variants (e.g., DRD4, SLC6A3) contribute modestly to heritability (1–3% per allele), supporting multifactorial etiology.	Elsayed et al., 2020	[26]
	Pharmacogenetic insights (e.g., ADRA2A, COMT) may guide personalized treatments, but clinical validation remains limited.	Ramsey et al., 2020; Brown, 2022	[27,28]
Key Themes	ADHD’s genetic architecture involves heritability, shared risks with comorbidities, and distinct familial subtypes, enabling targeted interventions.	Summary of studies	

Pharmacogenetics in ADHD

ADHD is a complex neurodevelopmental disorder that affects attention, impulse control, and executive functioning. Pharmacotherapy for ADHD typically involves stimulant medications, such as methylphenidate (MPH) and amphetamines, which target catecholamine pathways to modulate dopamine and norepinephrine activity [29]. However, the variability in patient responses to these medications has led to the exploration of pharmacogenetics, the study of how genetic variations influence drug response. This emerging field aims to personalize ADHD treatment, optimizing therapeutic outcomes and minimizing adverse effects by identifying genetic markers that influence drug efficacy.

Pharmacogenetics provides an opportunity to tailor ADHD treatment based on genetic profiles, particularly for genes involved in the dopaminergic and adrenergic systems. Current AACAP guidelines do not endorse pharmacogenetic testing for ADHD due to insufficient evidence [27]. Important pharmacokinetic genes, including CES1 and CYP2D6, influence drug metabolism but lack clinical validation [28]. Research on genetic variations in genes like SLC6A3, DRD4, ADRA2A, COMT, and others has advanced our understanding of how individual genetic differences can impact the effectiveness of ADHD medications [26]. By incorporating pharmacogenetic testing into clinical practice, it may be possible to predict which patients will respond best to specific treatments, thereby improving the overall management of ADHD.

SLC6A3 (Dopamine Transporter Gene)

The dopamine transporter gene (SLC6A3) plays a key role in ADHD treatment, as it encodes a protein responsible for dopamine reuptake, the primary target of stimulant medications like MPH and amphetamines [30]. Variations in the SLC6A3 gene, particularly the 10-repeat allele of a variable number tandem repeat (VNTR) in the 3′ untranslated region, have been associated with ADHD and differential responses to stimulant medications [31]. Studies suggest that individuals with the 10-repeat allele exhibit higher dopamine transporter densities, potentially influencing the neurobiological effects of stimulant medications [32]. Neuroimaging studies support these findings, showing that SLC6A3 polymorphisms play a role in modulating stimulant therapeutic effects [33]. PET imaging of MPH further revealed that it blocks over 50% of dopamine transporters, amplifying dopamine signaling, which enhances attention and motivation. The dopamine transporter gene (SLC6A3) encodes a presynaptic membrane protein responsible for dopamine reuptake, the primary target of stimulant medications. The 10-repeat allele of a VNTR polymorphism in the 3′ untranslated region has been associated with altered MPH efficacy. Meta-analyses of clinical trials indicate that the 10-repeat allele of SLC6A3 is associated with improved response to MPH, particularly in reducing hyperactivity. However, effect sizes remain small, underscoring the polygenic nature of ADHD pharmacogenetics [34].

DRD4 (Dopamine D4 Receptor Gene)

The DRD4 gene encodes a receptor involved in modulating dopamine signaling, particularly in brain regions related to attention and executive functions. The gene contains polymorphic variants such as D4.2, D4.4, and D4.7, with D4.7 being associated with increased ADHD risk [35]. Studies indicate that the D4.7 variant exhibits reduced receptor functionality, potentially contributing to altered dopaminergic signaling in ADHD. Moreover, the absence of functional interactions with the dopamine D2 receptor in D4.7 carriers may impair corticostriatal neurotransmission, which underpins ADHD symptoms [36]. Pharmacologically, DRD4 has been found to have a high affinity for clozapine, indicating its relevance in neuropsychiatric conditions, including ADHD. Understanding the role of DRD4 in ADHD can help personalize treatment strategies for patients, particularly when dealing with ADHD-related deficits in attention and executive functions. The DRD4 7-repeat allele has been associated with attenuated response to stimulants in earlier meta-analyses, particularly for inattentive symptoms [37]. Recent longitudinal data suggest that this variant may also influence medication adherence [36].

ADRA2A (Adrenergic α2A Receptor Gene)

The ADRA2A gene encodes the adrenergic α2A-receptor, which plays a significant role in ADHD pathophysiology by regulating catecholamine signaling in the prefrontal cortex (PFC) [38]. Variants in the ADRA2A gene, specifically the -1291 C>G polymorphism, have been linked to ADHD, especially the inattentive subtype. Studies suggest that individuals with the G allele of this polymorphism show better improvement in inattentive symptoms following MPH treatment. This suggests that the ADRA2A-1291 C>G polymorphism modulates treatment response, with the G allele facilitating MPH's beneficial effects on attention regulation [39]. A meta-analysis of six clinical trials confirmed that the ADRA2A-1291 G allele is associated with greater improvement in inattentive symptoms post-MPH treatment, with recent evidence suggesting this allele may also mitigate adverse effects [40].

COMT (Catechol-O-Methyltransferase)

The COMT enzyme regulates dopamine and noradrenaline levels in the PFC, a brain region crucial for attention and executive functioning [41]. A functional polymorphism in the COMT gene, the Val158Met variant, results in differential enzymatic activity. The Val allele is associated with increased enzyme activity and faster dopamine degradation, while the Met allele is linked to lower activity and slower dopamine clearance. This polymorphism has implications for ADHD, with the Val allele being associated with hyperactive-impulsive behaviors. Studies have shown that individuals with the Val allele may benefit more from MPH treatment, particularly in reducing hyperactivity and impulsivity [42]. Recent studies suggest that the COMT Val158Met polymorphism plays a modulatory role in stimulant response by influencing catecholaminergic neurotransmission. One study reported that children with ADHD who carry the Val/Val genotype exhibit significantly altered serum levels of dopamine and norepinephrine, which may impact both symptom presentation and response to pharmacotherapy [43]. Another investigation highlighted that COMT-related variations affect reward system neurodynamics and dopaminergic sensitivity, which may also contribute to individual differences in behavioral phenotypes relevant to ADHD [44].

DRD5 (Dopamine D5 Receptor Gene)

The DRD5 gene, a part of the dopaminergic system, plays a critical role in ADHD. Variations in a microsatellite marker upstream of DRD5 have been associated with ADHD, particularly the inattentive subtype [45]. Studies suggest that the 148-base pair allele is linked to ADHD susceptibility, reinforcing the idea that disruptions in dopaminergic signaling contribute to the disorder's clinical features [46]. The DRD5 gene, a member of the dopamine receptor family, has been implicated in the pathophysiology of ADHD through its role in modulating dopaminergic transmission. A specific 148-base pair microsatellite allele located upstream of the DRD5 gene has been consistently associated with increased ADHD susceptibility, particularly in individuals with the inattentive subtype. Recent pharmacogenetic evidence from randomized clinical trials indicates that individuals carrying this allele may exhibit enhanced clinical response to atomoxetine, suggesting a potential role for DRD5 genotyping in guiding non-stimulant therapy selection in ADHD management [47].

SLC6A2 (Noradrenaline Transporter Protein 1)

Regarding the SLC6A2 gene, which encodes the norepinephrine transporter (NET), genetic variation has been shown to influence both pharmacodynamics and treatment outcomes in ADHD [48]. Polymorphisms in this gene are particularly relevant for drugs that target norepinephrine reuptake, such as atomoxetine. A comprehensive meta-analysis involving 12 studies found that certain SLC6A2 variants were significantly associated with treatment response, particularly in hyperactive-impulsive presentations of ADHD, although the effect sizes remained modest (odds ratio ~1.2) [49]. Variants in this gene have been explored in relation to ADHD and treatment response, particularly to MPH. Studies indicate that individuals with specific polymorphisms in SLC6A2 may exhibit different responses to MPH, particularly with regard to hyperactive-impulsive symptoms [50]. This highlights the potential of pharmacogenetic testing to predict treatment efficacy based on SLC6A2 genetic variations.

Pharmacogenetics offers a promising new approach to ADHD treatment, providing insights into how genetic variations influence medication response. By identifying genetic markers such as those in SLC6A3, DRD4, ADRA2A, COMT, DRD5, and SLC6A2 (Table 2), clinicians may be able to tailor ADHD treatment to individual patients, improving therapeutic outcomes and minimizing adverse effects. The integration of pharmacogenetic testing into clinical practice represents a significant step toward personalized medicine in ADHD management, enhancing the precision of treatment strategies.

**Table 2:** Summary of the role and variants in ADHD along with its implications ADHD: Attention-Deficit/Hyperactivity Disorder

Genetic Marker	Gene Function/Role	Polymorphisms/Variants	Associated ADHD Subtype	Study Findings	Pharmacogenetic Implications for Treatment
SLC6A3	Dopamine transporter regulates dopamine reuptake, a key target of stimulant medications (MPH, amphetamines).	10-repeat allele (VNTR) in the 3′ untranslated region	General ADHD (with stimulant response)	10-repeat allele linked to higher dopamine transporter density, affecting stimulant medication response [31,32].	Higher transporter density may lead to stronger effects from stimulants like MPH, aiding in tailoring stimulant medication use.
DRD4	Dopamine D4 receptor modulates dopamine signaling related to attention and executive functions.	D4.2, D4.4, D4.7 variants	ADHD, particularly attention deficits	D4.7 variant associated with reduced receptor functionality, leading to altered dopaminergic signaling [36].	D4.7 may impair dopamine signaling, requiring alternative or adjusted ADHD treatment strategies, especially for executive function deficits.
ADRA2A	Adrenergic α2A receptor regulates catecholamine signaling in the prefrontal cortex (PFC).	-1291 C>G polymorphism	Inattentive subtype of ADHD	G allele associated with better improvement in inattentive symptoms with MPH treatment [39,40].	Individuals with G allele may have a better response to MPH for attention-related symptoms, facilitating more precise treatment.
COMT	Catechol-O-methyltransferase regulates dopamine and norepinephrine levels in the PFC.	Val158Met polymorphism	Hyperactive-impulsive subtype of ADHD	Val allele linked to faster dopamine degradation, leading to more hyperactivity and impulsivity [42,43].	Val allele carriers may benefit from MPH for reducing hyperactivity and impulsivity, suggesting a targeted approach in pharmacotherapy.
DRD5	Dopamine D5 receptor, part of the dopaminergic system.	148-base pair allele (microsatellite marker)	Inattentive subtype of ADHD	148-base pair allele linked to ADHD susceptibility and attentional deficits [46,47].	DRD5 variant suggests a predisposition to inattentive ADHD, potentially guiding medication choices for attention-related symptoms.
SLC6A2	Norepinephrine transporter regulates norepinephrine levels.	Specific polymorphisms	Hyperactive-impulsive symptoms of ADHD	Variants in SLC6A2 linked to differential MPH response, particularly for hyperactive-impulsive symptoms [50].	Polymorphisms in SLC6A2 may predict the efficacy of MPH, especially for hyperactive-impulsive ADHD symptoms, aiding in pharmacogenetic testing.

Despite growing evidence linking genetic variations to differential treatment response in ADHD, current clinical guidelines do not recommend routine pharmacogenetic testing for ADHD management. This caution stems from the challenges of translating pharmacogenomic findings into clinical practice, including polygenic complexity, variability in study outcomes, and the absence of large-scale, prospective RCTs. Recent expert consensus highlights the need for standardized testing protocols and better integration of pharmacogenomics into psychiatric care pathways while emphasizing the importance of interdisciplinary collaboration to interpret test results meaningfully and ethically [51]. Furthermore, real-world implementation studies underscore that the incorporation of pharmacogenomics into team-based care models, although promising, requires addressing logistical, economic, and training-related barriers before widespread adoption can be realized [52]. Future research should focus on validating clinical utility through cost-effectiveness analyses, implementation science, and multicenter trials to establish robust, evidence-based guidelines for personalized ADHD pharmacotherapy.

Biomarkers in ADHD

Monoaminergic Transmission Pathways in ADHD

The dopaminergic system is central to understanding the pathophysiology of ADHD, as evidenced by abnormalities in dopamine synthesis, transporter function, and receptor-mediated processes [53]. These alterations underpin many symptoms of ADHD and influence therapeutic responses. While studies have extensively explored the dopaminergic pathway, a stronger connection between findings across neuroimaging, genetic, and pharmacological studies could provide deeper insights into its role as a biomarker [54].

Alterations in Dopamine Synthesis and Turnover

Ludolph et al. investigated dopaminergic dysfunction in young adults with ADHD using FDOPA PET scans. Their findings revealed reduced dopamine influx rates (Ki) in regions such as the bilateral putamen, amygdala, and dorsal midbrain in untreated ADHD patients compared to healthy controls. Interestingly, specific patterns, such as reduced Ki in the left putamen and right dorsal midbrain and increased Ki in the left amygdala and right anterior cingulate cortex, suggested the presence of compensatory mechanisms [55]. These observations align with those of Forssberg et al., who found decreased utilization of L-[11C]-DOPA in the striatum and midbrain of adolescents with ADHD. This reduced dopamine synthesis correlated with symptoms of inattention, supporting the idea that dopamine metabolism deficits in specific subcortical regions are linked to cognitive impairments in ADHD [56]. Together, these studies underscore the importance of dopamine turnover and regional dopamine synthesis in ADHD.

Dopamine Transporter (DAT) Dysfunction

Volkow et al. used PET imaging to study dopamine transporter (DAT) availability in drug-naïve adults with ADHD. The study revealed reduced DAT levels in the left caudate and nucleus accumbens, with no significant differences in the putamen. A strong association between inattention scores and putamen DAT levels was observed in both ADHD and control groups, highlighting DAT's regulatory role in attention [57]. These findings are consistent with those of Krause, who linked increased DAT availability to untreated ADHD patients, further associating it with the DAT1 gene polymorphism. Krause also noted that MPH treatment normalized elevated DAT levels, suggesting that DAT availability might predict treatment response. Substances like nicotine and zinc, which lower DAT availability, were also found to alleviate ADHD symptoms, adding a layer of pharmacological complexity to DAT's role in ADHD [58].

Dysregulated Dopamine Efflux and Receptor Interactions

Bowton et al. examined the interplay between dopamine D2 autoreceptors and DAT function in ADHD. They found that an ADHD-associated DAT variant (Ala559Val) facilitated anomalous dopamine efflux, similar to the effects of psychostimulants. This efflux was driven by D2 receptor activation and CaMKII-mediated phosphorylation, suggesting a dysregulated signaling network that exacerbates dopamine-related dysfunctions [59]. These findings complement those of Volkow et al. by emphasizing how DAT availability and receptor interactions might contribute to ADHD’s clinical variability [57].

Norepinephrine and Dopamine Modulation

Bymaster et al. explored the effects of atomoxetine, a norepinephrine transporter (NET) inhibitor, on dopamine levels. PET imaging reveals that atomoxetine, a selective NET inhibitor, increases prefrontal norepinephrine levels by 300% by blocking its reuptake. This enhances norepinephrine signaling, improving attention and impulse control, which is beneficial in ADHD treatment [60]. This region-specific modulation highlights atomoxetine's efficacy in enhancing attention and executive function, providing an alternative to psychostimulants like MP, which increase dopamine in reward circuits and carry a higher abuse potential. The distinct mechanism of action of atomoxetine supports the idea that monoaminergic modulation can have tailored therapeutic outcomes in ADHD. Collectively, these studies elucidate various aspects of dopaminergic and monoaminergic dysfunctions in ADHD, emphasizing the interplay between dopamine synthesis, DAT availability, receptor regulation, and pharmacological modulation. Ludolph et al. and Forssberg et al. provide complementary insights into dopamine synthesis deficits and regional dysfunctions, correlating these with ADHD symptoms [55,56]. Volkow et al. and Krause underscore DAT's central role, linking its availability to genetic polymorphisms, symptomatology, and treatment outcomes [57,58]. Finally, Bowton et al. and Bymaster et al. delve into the molecular mechanisms underlying dopamine efflux and the therapeutic implications of targeting monoaminergic pathways [59,60]. Despite these advances, gaps remain in connecting neuroimaging, genetic findings, and treatment outcomes to establish dopaminergic dysfunction as a robust biomarker for ADHD. Future research should aim to standardize methodologies, integrate genetic and neuroimaging data, and explore longitudinal changes to strengthen the case for dopaminergic pathways as biomarkers for ADHD diagnosis and treatment monitoring.

Elemental Biomarkers

ADHD, characterized by inattention, hyperactivity, and impulsivity, has been linked to imbalances in essential elements such as magnesium (Mg), zinc (Zn), and copper (Cu) [61]. These elements, often acting as enzymatic cofactors, can influence neuronal activity and potentially impact ADHD symptomatology. While existing studies highlight their importance, there remains a need to synthesize findings into a cohesive narrative to explore their utility as diagnostic biomarkers for ADHD.

Deficiencies in Magnesium, Zinc, and Copper

Recent systematic reviews synthesized global evidence on ADHD biomarkers and concluded that elemental markers like magnesium (Mg), zinc (Zn), and copper (Cu) lack sufficient sensitivity and specificity (AUC <0.8) for diagnostic use, despite replicated associations in some studies [62]. For instance, a case-control study in Eastern India found altered Cu and Zn levels in hair and urine samples of children with ADHD, highlighting regional variations in elemental imbalances. However, this study had limitations, including a small sample size and a lack of validation against clinical outcomes, underscoring the need for cautious interpretation [63].

Individual studies have reported mixed findings. One analysis found significant Mg (65%), Zn (60%), and Cu (70%) deficiencies in serum and hair samples of children with ADHD compared to healthy controls, with Mg and Zn deficiencies correlating strongly with symptom severity [64]. Another study demonstrated that supplementation with magnesium and vitamin B6 positively influenced social behaviour and reduced excitability in children, providing mechanistic insights into how Mg-B6 regulates central nervous system activity [65]. Zinc’s role in cognitive functions was highlighted by Arnold et al., who reported that serum Zn levels in the lowest 30% of the reference range correlated with inattention severity but not hyperactivity-impulsivity [66]. However, contradictions exist: elevated serum Mg levels were observed in some ADHD cohorts, alongside altered lipid profiles (e.g., increased HDL), pointing to complex metabolic interactions and bidirectional relationships between Mg levels and ADHD [67].

These inconsistencies, coupled with the conclusion of Cortese et al. that no elemental biomarker meets clinical utility thresholds, emphasize that Mg, Zn, and Cu should be framed as potential research targets rather than diagnostic biomarkers [68]. Further large-scale, longitudinal studies are needed to clarify their role in ADHD pathophysiology and therapeutic strategies.

Therapeutic Potential of Mineral Supplementation

The therapeutic implications of elemental imbalances are underscored by a meta-analysis of 12 studies confirming significantly lower Mg levels in ADHD children across plasma, serum, and hair samples [69]. These findings were corroborated by another study that reported sustained symptom improvement with Mg-B6 supplementation. Interestingly, ADHD symptoms reemerged upon cessation of supplementation, reinforcing the active role of Mg in symptom modulation [70].

Exploring the effects of stimulant medications on mineral levels, one study found that stimulant use increased plasma Mg levels while altering the calcium-to-magnesium ratio. These shifts may influence both therapeutic outcomes and side effects, suggesting the importance of monitoring mineral levels during treatment [71]. Furthermore, salivary Mg levels were proposed as a potential diagnostic biomarker. Reduced salivary Mg levels, coupled with elevated oxidative stress markers, showed over 90% sensitivity and specificity for diagnosing ADHD. This aligns with serum and hair analysis studies, providing a non-invasive biomarker option that ties Mg deficiency to ADHD's oxidative stress-related pathophysiology [72].

Despite promising findings, a systematic review noted the lack of conclusive evidence in mineral supplementation trials for ADHD. This highlights the need for rigorously designed studies to establish standardized treatment protocols and validate elemental levels as diagnostic biomarkers [73].

The collective evidence highlights a multifaceted relationship between elemental imbalances and ADHD. Mg appears to influence hyperactivity, aggression, and attention deficits, while Zn specifically affects inattention [74]. Although the role of Cu remains ambiguous, its interactions with other cofactors warrant further study. Contradictory findings emphasize the complexity of mineral interactions and their metabolic underpinnings in ADHD. By integrating insights from various studies, a clearer picture emerges. Mg and Zn deficiencies are not merely markers but active contributors to ADHD's pathophysiology. Their potential as diagnostic biomarkers and as therapeutic targets positions them as critical areas for future research [75].

Immune and Inflammatory Biomarkers in ADHD

ADHD is a neurodevelopmental disorder that significantly affects individuals' educational, social, and emotional well-being. While ADHD's etiology remains elusive, emerging evidence suggests that immune-inflammatory mechanisms play a pivotal role in its pathophysiology, potentially bridging the gap between genetic predispositions and environmental triggers [76].

Maternal Inflammation as a Risk Factor

Maternal inflammation during pregnancy has been explored as a potential early predictor of ADHD risk in offspring [77]. Initial studies reported that elevated maternal cytokine levels, including interleukin-6 (IL-6) and tumor necrosis factor-α (TNF-α), during the third trimester were associated with ADHD symptoms in children aged 4-6 years (β = 0.53, p = 0.006), independent of familial ADHD history [78]. These findings suggested that maternal inflammation might mediate prenatal distress effects on ADHD outcomes, positioning IL-6 as a candidate biomarker for early intervention.

However, subsequent research has yielded inconsistent results. A meta-analysis of risk of ADHD associated with maternal infection during pregnancy found no significant association between maternal IL-6 levels and child ADHD after adjusting for genetic and environmental confounders [79]. Similarly, earlier studies highlighted the role of shared genetic factors in explaining the link between maternal inflammation and ADHD, challenging the assumption of a direct causal relationship [80]. A systematic review further concluded that maternal inflammatory markers, including IL-6 and TNF-α, lack sufficient specificity and sensitivity (AUC <0.8) for clinical use in ADHD diagnosis or risk prediction [81]. While these findings underscore the complexity of maternal immune activation in ADHD etiology, they caution against premature application of inflammatory biomarkers in clinical settings.

Gene Expression Signatures in Immune Pathways in ADHD

Expanding beyond maternal inflammation, immune-related gene expression offers novel insights into ADHD's etiology. A weighted gene co-expression network analysis (WGCNA) across neurodevelopmental and psychiatric disorders identified distinct immune signatures in ADHD [82]. Hub genes, such as YY1 and PARP14, emerged as key differentiators, particularly in adult ADHD. These findings underscore the interplay between immune pathways and ADHD symptomatology, linking immune dysregulation to ADHD's neurobiological underpinnings [83]. This study aligns with other research that highlighted immune pathways' contributions to psychiatric phenotypes, emphasizing shared biological mechanisms [84]. Furthermore, studies demonstrated genetic overlaps between ADHD and related disorders, suggesting that immune-related biomarkers could facilitate subtype differentiation and cross-disorder diagnosis [83].

Inflammatory Biomarkers as Diagnostic Tools

Advancements in ADHD research propose cytokines, particularly IL-6 and TNF-α, as potential biomarkers. Systematic reviews highlight inconsistent evidence for IL-6 and TNF-α in ADHD, with some studies reporting elevated IL-6 levels and others showing no significant changes, while TNF-α findings remain conflicting [76,81]. These biomarkers may illuminate biological mechanisms and offer non-invasive tools, though current evidence does not support their clinical utility for diagnosis [85]. For instance, while IL-6 has been linked to ADHD symptoms in some cohorts [86], immune hub genes emphasize exploratory connections between immune-related gene expression and ADHD subtypes [87]. Together, these studies suggest avenues for research into inflammatory biomarkers, though further validation is required before clinical integration [88].

The interaction between genetic predispositions and environmental factors, such as prenatal inflammation, underlines the need for multifaceted approaches. Although polygenic risk scores did not correlate significantly with gene expression, studies highlight how environmental influences shape immune-related gene expression [89]. These findings align with integrative strategies combining genetic, immune, and environmental data. Cumulatively, these findings contribute to understanding ADHD’s pathophysiology, though inflammatory biomarkers are not yet validated for clinical application [88].

Salivary Biomarkers

Salivary biomarkers have emerged as a promising and innovative tool for the diagnosis and understanding of ADHD. Unlike traditional diagnostic techniques, which depend on behavioral assessments and subjective reports, salivary biomarkers offer objective, non-invasive insights into the physiological and biochemical mechanisms underlying ADHD [90]. Biomarkers such as salivary cortisol and alpha-amylase (sAA) provide unique insights into the dysregulation of the hypothalamic-pituitary-adrenal (HPA) axis and the sympathetic nervous system (SNS), which are known to play a role in ADHD [91]. These biomarkers enable the exploration of stress responses, immune-inflammatory activity, and adrenergic signaling, offering a clearer picture of the physiological disruptions associated with ADHD. sAA has been found to be significantly elevated in children with ADHD, reflecting heightened SNS activity. This aligns with earlier studies linking sAA to stress-induced dysregulation, making it a reliable marker for ADHD-related stress responses [90].

In contrast, salivary cortisol, a key HPA axis biomarker, shows mixed patterns in ADHD. Studies comparing ADHD and neurotypical individuals report inconsistent findings [85]. A study found no significant differences in baseline cortisol levels, whereas others observed blunted stress responses in individuals with ADHD [92]. These discrepancies may reflect heterogeneity in ADHD subtypes, comorbidities, or methodological variations in cortisol measurement. This emphasizes the need to explore multiple biomarkers to understand the nuanced dysregulation of both the HPA axis and SNS in ADHD. Additionally, the study revealed elevated levels of salivary immunoglobulins, particularly secretory IgA (sIgA), in children with ADHD. This suggests a potential link between immune system activity and stress responses in ADHD, reflecting broader systemic involvement in the disorder beyond neurobehavioral symptoms [93].

Increased sIgA levels, which are associated with oral mucosal hypersensitivity, could also indicate heightened susceptibility to infections, providing a new perspective on ADHD pathophysiology. These advancements in salivary biomarker research mark a shift towards non-invasive, personalized diagnostic approaches for ADHD. By incorporating biomarkers like sAA, cortisol, and immunoglobulins, researchers can move beyond behavioral assessments to achieve more precise physiological profiling. Future studies should explore additional biomarkers, such as chromogranin A (CgA) and cytokines like IL-6, to further unravel the immune-inflammatory pathways involved in ADHD, enhancing diagnostic accuracy and therapeutic monitoring [81,94]. This holistic integration of salivary biomarkers into ADHD research exemplifies a novel, cutting-edge approach to diagnosis and treatment, offering accessible and repeatable tools for clinical practice.

Electroencephalogram (EEG) Markers in ADHD

EEG markers are pivotal in understanding the neurophysiological underpinnings of ADHD. EEG measures the brain's electrical activity through frequency bands, including delta, theta, alpha, and beta, reflecting various cognitive and behavioral states. In ADHD, these markers highlight distinctive patterns, such as elevated theta activity and reduced beta activity, indicative of impaired cortical arousal and executive functioning. One of the most notable EEG markers is the theta/beta ratio (TBR), which has been widely studied for its diagnostic potential [95]. ADHD children often exhibit a higher TBR compared to healthy controls, a finding linked to attentional deficits and hyperactivity [96,97]. These markers not only aid in diagnosis but also offer insights into the heterogeneity of ADHD and the potential for personalized treatment strategies. By providing objective measures of brain function, EEG markers represent a valuable advancement in ADHD research, complementing traditional behavioral assessments and paving the way for innovative diagnostic and therapeutic approaches. Studies have consistently shown that children with ADHD exhibit increased absolute and relative theta and delta activity, along with reduced alpha and beta activity, compared to healthy controls [98]. This has been confirmed across multiple age groups, including children, adolescents, and adults, suggesting a persistent neurophysiological profile associated with ADHD [99]. These patterns indicate slower cortical activity, particularly in regions responsible for executive functions and attention regulation. The TBR has emerged as a prominent EEG marker for ADHD diagnosis. TBR, defined as the ratio of theta (4-8 Hz) to beta (13-21 Hz) power, has shown increased values in ADHD populations, especially in children [98]. Monastra et al. reported that ADHD children exhibit a TBR three times higher than healthy controls, with a sensitivity of 86-90% and specificity of 94-98% [100]. While TBR is predominantly measured at the Cz electrode, studies suggest that its reliability varies with age and electrode positioning, requiring further refinement for clinical applications. The use of absolute and relative EEG power spectra has revealed critical developmental differences between ADHD and control groups [101]. For instance, while ADHD children exhibit heightened absolute slow wave activity, adults with ADHD often show normalization in certain bands, reflecting either maturation or compensatory mechanisms [102]. However, inconsistencies in findings, such as variations in beta activity and TBR, highlight the heterogeneity of ADHD and the influence of comorbidities [103]. Recent advancements in EEG studies have expanded their application beyond diagnosis to treatment monitoring. Quantitative EEG (qEEG) and biofeedback approaches now offer real-time interventions to regulate cortical activity, supporting personalized treatment strategies [104]. Additionally, multicenter studies have validated the use of EEG markers in predicting treatment outcomes, demonstrating the potential of EEG as a non-invasive tool for optimizing therapeutic decisions [105].

Despite its advancements, challenges remain in standardizing EEG protocols and addressing variability across populations. EEG has variable diagnostic accuracy, with an accuracy of 85% and a sensitivity of 63% [106]. While EEG effectively detects abnormalities in most true cases, its lower specificity increases the risk of false positives, potentially leading to misdiagnosis. Challenges remain in standardizing EEG protocols and addressing variability across populations. The development of age-specific norms and robust classification algorithms will be crucial for translating EEG findings into routine clinical practice. Nonetheless, EEG-based diagnostics represent a significant leap forward in understanding ADHD, offering objective, physiological insights that complement traditional behavioral assessments. Nonetheless, while EEG studies offer valuable insights into ADHD-related neurophysiological patterns, a recent systematic review concluded that EEG-based diagnostic tools have not yet been independently validated or replicated. As such, they are not ready for routine clinical use, despite their potential to complement behavioral assessments [107].

Artificial intelligence in ADHD

Traditional diagnostic and therapeutic methods are often subjective, time-consuming, and inaccessible in various regions, creating a gap in efficient care delivery. AI has emerged as a transformative tool in ADHD research, offering innovative solutions for diagnosis, treatment, and therapy personalization [108]. AI leverages advanced data analytics, machine learning, and deep learning algorithms to analyze complex datasets, enabling precise diagnosis and tailored interventions. This review highlights recent advancements in AI applications for ADHD, showcasing its potential to revolutionize ADHD care. AI is revolutionizing ADHD research and care by offering objective diagnostics through quantifiable markers like eye movements, EEG patterns, and pupil dynamics, while tools such as Pepper robots and web-based applications enhance scalability and accessibility, particularly in underserved regions [15]. By integrating patient-specific data, AI enables personalized therapy, improving treatment efficacy and cost-efficiency through hybrid models and wearable technologies.

AI-based diagnostic and therapeutic interventions

Socially assistive robotics, exemplified by the Pepper humanoid robot, enhances therapeutic engagement in children by integrating interactive exercises with real-time emotional monitoring. This technology reduces the fear of human interaction and promotes individualized care, serving as a reliable intermediary in ADHD diagnostics and therapy [109]. For adults, hybrid AI models have demonstrated significant potential, such as the UK-based study that achieved 93.61% diagnostic accuracy by combining machine learning with interpretable knowledge-based algorithms. These models emulate clinical decision-making, reduce dependency on expensive diagnostic interviews, and address comorbidities like anxiety and bipolar disorder, thereby making ADHD diagnosis more scalable and accessible [110].

Trends in automated ADHD detection underscore the versatility of AI, with applications spanning brain imaging, physiological signals, performance tests, and wearable devices. These tools address diagnostic delays caused by clinician shortages and comorbid conditions, offering a pathway to faster, more accurate diagnoses [111]. Non-invasive technologies, such as tablet-based AI eye-tracking systems, are promising tools to identify ADHD-specific patterns through tasks like prosaccade and antisaccade trials, offering scalable and accessible solutions particularly suitable for children but they lack real-world validation due to cost barriers [112]. Similarly, AI-driven motor information analysis using EEG data achieves diagnostic accuracies of up to 98.21%, revealing unique motor and cognitive patterns associated with ADHD and paving the way for personalized therapeutic strategies [113].

Recent advances have shown that pupil dynamics, when analyzed through machine learning frameworks, can serve as objective and interpretable biomarkers for ADHD. A recent study demonstrated that temporal features of pupil responses enabled accurate classification of ADHD versus controls, achieving strong diagnostic performance with interpretable AI models. Beyond accuracy, their framework emphasized transparency of decision-making, a critical step toward clinical acceptance. These findings position AI-driven pupil dynamics as a cost-effective, scalable, and physiologically grounded approach to ADHD diagnosis, with particular promise for enhancing accessibility in resource-limited settings [114]. For behavioral therapy, AI-powered implementation models incorporating tools like motivational interviewing and integrity monitoring have improved community-based care for adolescents. These tools enhance therapy adherence, clinician support, and intervention efficiency through dashboards and AI-generated feedback, optimizing community-level ADHD care [115].

Collectively, these advancements highlight the interdisciplinary potential of AI in ADHD care, integrating robotics, physiological data analysis, and hybrid algorithms. By combining diverse technologies such as socially assistive robots, wearable devices, and EEG-based diagnostics, AI not only enhances diagnostic precision but also personalizes therapy, ultimately making ADHD care more efficient, accessible, and impactful [114,115].

## Conclusions

Advances in genetics, neuroimaging, AI, and pharmacogenetics are reshaping ADHD diagnostics and treatment, offering the potential for more personalized and objective care. Social robotics, wearables, and nutritional interventions complement traditional therapies, improving engagement and outcomes. However, significant barriers remain, including biomarker heterogeneity, limited validation, high costs, and lack of cost-effectiveness. Current tools like AI-based diagnostics and pharmacogenetic testing show promise but are not yet ready for routine clinical use. Future research should emphasize large-scale, longitudinal, and multimodal approaches that integrate genetic, behavioral, and physiological data. As technologies become more validated, affordable, and accessible, especially in low-resource settings, they may enable equitable, personalized, and holistic ADHD care.
